# Challenges in access to health services and its impact on quality of life: a randomised population-based survey within Turkish speaking immigrants in London

**DOI:** 10.1186/1477-7525-10-11

**Published:** 2012-01-26

**Authors:** Kenan Topal, Erhan Eser, Ismail Sanberk, Elizabeth Bayliss, Esra Saatci

**Affiliations:** 1 Department of Family Medicine, Pamukkale University Faculty of Medicine, Denizli 20200, Turkey; 2 Department of Public Health, Celal Bayar University Faculty of Medicine, Manisa 45040, Turkey; 3 Department of Psychological Counselling and Guidance, Cukurova University Faculty of Education, Adana 01130, Turkey; 4 Executive Director of Social Action for Health, 192 Hanbury Street, London E1 5HU, UK; 5 Department of Family Medicine, Cukurova University Faculty of Medicine, Adana 01130, Turkey

**Keywords:** Turkish immigrants, health services, accessibility, Quality of Life, well-being

## Abstract

**Background and aim:**

There are a significant number of Turkish speaking immigrants living in London. Their special health issues including women's health, mental health, and alcohol and smoking habits has been assessed. The aim of this study was to explore the ongoing challenges in access to health care services and its impact on Quality of Life of immigrants.

**Material and methods:**

This cross-sectional population-based study was conducted between March and August 2010 with Turkish immigrants (n = 416) living in London. Of these, 308 (74%) were Turkish and 108 (26%) were Turkish Cypriots. All healthy or unhealthy adults of 17-65 years of age were enrolled. A structured questionnaire with 44 items in five subcategories and 26-items WHOQOL BREF were used.

**Results:**

Mean duration of stay for Turkish Cypriots (26.9 ± 13.9 years) was significantly longer than Turkish immigrants (13.3 ± 7.5) (p < 0.001). Turkish immigrants (n = 108, 36.5%) need interpretation more often when using health services than Turkish Cypriots (n = 16, 15%) (p < 0.001). Multivariate analyses suggested significant effects of older age, non-homeownership, low socioeconomic class, poor access to health services, being ill, poor community integration and being obese on physical well-being and also significant effects of low income and poor community integration on perceived overall Quality of Life (WHOQOL) of the participants.

**Conclusions:**

The results of this study demonstrate how the health and well-being of members of the Turkish speaking community living in London are affected by social aspects of their lives. Providing culturally competent care and interpretation services and advocacy may improve the accessibility of the health care.

## Introduction

The growing scope of migratory movements all over the world raises specific health questions in both sending and receiving countries. Migrants are particularly vulnerable to health problems [1]. The biomedical and biopsychosocial dimensions of migration will possibly pose new and more difficult challenges to those who move, those they leave behind and those who host them in receiving societies [2]. Migrants often experience other life transitions, such as occupational and socioeconomic changes and social network alterations after physical relocation [3]. Significant number of Turkish speaking immigrants is living in London [4]. Like other vulnerable groups, they face with various obstacles in access to health care services. Studies draw attention to cultural and linguistic barriers and a lack of information or understanding about how the healthcare system works in the United Kingdom (UK) [5-7].

There are a number of studies focusing on special health issues including women's health, mental health, and alcohol and smoking habits among Turkish Speaking Communities (TSCs) [8-12]. On the other hand, little attention has been paid to Quality of Life (QoL) of TSCs with validated scales up-to-date. The World Health Organization (WHO) defines QoL as an individual's perception of their position in life in the context of the culture and value systems in which they live. WHOQOL BREF is a QoL assessment tool and was developed by the WHO. It is a cross-culturally valid assessment within four major domains: physical and psychological well-being, social relationship and satisfaction with environment [13-16].

TSCs have been a quite big language community in the UK since the early 1950s. The communities came originally from Cyprus and Turkey and have different immigration patterns. The Turkish Cypriots were the first to arrive in England and can be traced to the 1920s. Increased numbers arrived in the 1940s and late 1950s. A large wave from Cyprus came in the 1960s after the island became independent. Another large wave came as a result of the Turkey's intervention in 1974. This group's ties to Britain are stronger, as Cyprus was a British colony until 1960. Migration from mainland Turkey to Britain due to various economic and political reasons began in the early 1970s and followed 1980s and 1990s. There are smaller communities scattered around Britain but the majority of them live in and around London. They are working and living in the same areas and involved in similar economic, political, social and cultural activities [4].

The aim of this study was to explore the ongoing challenges when accessing to health services and to assess its impact on the QoL. Sharing the results with public authorities will hopefully improve the well-being of TSCs in terms of reaching and using health care services.

## Subjects and methods

This cross-sectional population-based study was conducted between March and August 2010 with Turkish immigrants (n = 416) living in London; Turkish Immigrants (Group A, n = 308, 74%) and Turkish Cypriots (Group B, n = 108, 26.0%). All healthy or unhealthy adults of 17-65 years of age were enrolled. Inclusion criteria were as follows: Being ≥ 17 years of age, giving written consent, living in the UK at least for one year.

TSCs are heavily concentrated in the London boroughs of Haringey, Enfield, Edmonton, Waltham Forest, Islington and Hackney. There are a large number of Turkish enterprises and Turkish food markets, kebab houses and coffee shops especially in Hackney and Haringey. Many Turkish community centres settled in these regions. They help Turkish speaking immigrants overcome barriers to education, employment, health and other problems. Advice on benefits, housing, immigration and nationality is provided. There is also English for Speakers of Other Languages (ESOL), computer, art, dancing and drama courses. Some of them give educational support for primary and secondary school children. Most of them also run a luncheon club for the elderly.

### Promotion and advocacy activities

Social Action for Health (SAfH) is a community development charity based in East London and supported this survey. SAfH works alongside marginalized local people and links community groups for mutual benefit, help communities to organise and encourage local people to take more control of their lives and their health and well-being. A launch meeting with a conference for research project was hosted by SAfH at Hackney office at May 21^st ^2010. This was attended by representatives from local Turkish community centres, health care workers, health advocates and carers, and other community members. The research project was announced in seven local community-based newspapers, broadcasted in a radio and television interviews. The participants in this study were reached using the database of SAfH and Turkish community centres working with together. Many organizations and enterprises and 13 community centres joined the study.

### Questionnaires

A structured questionnaire with 44 items in five subcategories was completed face-to-face. These subcategories are as follows: Sociodemographic, cultural and financial characteristics (9 items), migration patterns and issues of migration (7 items), work life (7 items), objective health status and access to health services (14 items) and health promotion or risk behaviours (7 items). Then, 26-item WHOQOL BREF scale which was developed by the World Health Organization (Additional file 1) was completed. It is a QoL assessment tool which is currently scored in four domains: Physical health (7 items), psychological well- being (6 items), social relationship (3 items) and satisfaction with the environment (8 items). Each item is rated on a 5-point Likert scale and the domain scores are transformed to 4-20 to enable comparisons. Domains are not scored where 20% of items or more are missing, and are unacceptable where two or more items are missed except for the Environment Domain allowing two missing items. Four types of 5-point Likert interval response scales were used in the WHOQOL-BREF. Items inquire 'how much', 'how completely', how often', 'how good' or 'how satisfied' the respondent felt in the last two weeks; different response scales are distributed across the domains. [17] The Turkish version of the WHOQOL BREF (TR) was reliable and valid [15].

### Sampling and data collection

There is no accurate population database on the size of the TSCs but reliable estimates suggest that the population may be around 340.000-360.000 [12]. The sample size calculation was set up a 95% confidence interval with significance level of p < 0.05. The estimated size of sample is 384. Sample selection was done by a mixed method of randomised cluster sampling and snowball sampling approaches because of unavailability of the entire list of Turkish inhabitants in London. The available but restricted list of Turkish population supplied by SAfH was used as a database for the selection of one person for each of the sampling clusters. A total of 39 clusters and one person for each cluster were identified. These 39 persons were selected by random selection from the list and each was asked to invite 10 Turkish friends for the study. As a result, 416 Turkish speaking persons were involved. The target sample size was increased from 384 to 416 by the invitation of more than 10 persons for each cluster in some clusters to increase the power of the study. The questionnaires were applied by the principal investigator and the three trained bilingual interviewers supported by SAfH between May-July 2010.

The questionnaire was prepared both in English and Turkish and WHOQOL-BREF or the Turkish version WHOQOL-BREF (TR) were used [15]. Participants were interviewed either in English or Turkish. All participants gave written consent. Consent forms were secured at SAfH Hackney Office Data was installed in August 2010 and consent forms and questionnaires were kept locked until the end of December 2010 and then shredded.

### Statistical Analyses

Descriptive statistics were used to summarize data for sociodemographic characteristics, migration patterns and issues of migration, objective health status and accessibility to health services. The dependent variables (i.e. domains of the WHOQOL) scores were dichotomized via the median values. "Health services access questions" were composed in order to generate a "health services access composite index score". This index score was categorized into three broad categories for the relevancy to the logistic regression models. Chi square tests and independent samples t-test were used in the bivariate analyses. Logistic regression analysis (LRA) was used to assess the final effects of the independent variables on the dependent variables. Adjusted Odds ratios were used as final measures of causality. For all statistical tests, *p *values less than 0.05 were considered as significant.

## Results

The sociodemographic characteristics of the participants are presented in Table 1. Of participants, 50.5% were male; the mean age was 38.9 ± 1.1 years (range = 17-65). Participants in Group B were significantly older than the participants in Group A (p < 0.001). The educational status of Group B was higher than that of Group A (41.7% vs. 34.1% graduated from secondary school) (p < 0.001). There was no significant difference between the two groups in terms of number of children (p > 0.05) however the families in Group B were more likely to have adults than those in Group A (25% vs. 9.7%) (p < 0.001). Unemployment rate of Group A (32.1%) seems to be higher than that of Group B (6.5%) and Group B was more crowded at 'middle management' (43,5%) than Group A (26,0%), (p < 0.001). More than half of participants in Group B (58.3%) stated that their income was at 'average levels of society' and20.4% at 'a little above average'; the rates of Group A were 35.4% and 11.7%, respectively, for the same variables (p < 0.001) (Table 1).

**Table 1:** Sociodemographic characteristics of Turkish Speaking Communities.

Sociodemographic characteristics		Turkish Immigrants (Group A) (n = 308)		Turkish Cypriots (Group B) (n = 108)		Total	
**Age (mean ± SD) (years)**		36.9 ± 9.5		44.6 ± 1.4		38.9 ± 1.1	

**Gender**	Male	156	50.6	54	50.0	210	50.5
	
	Female	152	49.4	54	50.0	206	49.5

**Date of birth**	1945-1960	36	11.7	49	**45.4^†^**	85	20.4
	
	1961-1970	79	25.7	26	24.1	105	25.2
	
	1971-1980	127	41.2	9	8.3	136	32.8
	
	1981-1993	66	21.4	24	22.2	90	21.6

**Education**	Illiterate	8	2.6	7	6.5	15	3.6
	
	Primary school	95	30.8	26	24.1	121	29.1
	
	Secondary school	105	34.1	45	**41.7^†^**	150	36.1
	
	High school	100	32.5	30	27.8	130	31.2

**Household type: adults**	1 adult	72	23.4	16	14.8	88	21.2
	
	2 adults	177	57.5	52	48.1	229	55.0
	
	3 adults	30	9.7	27	**25.0^†^**	57	13.7
	
	4 adults	18	5.8	10	9.3	28	6.7
	
	5 or more adults	11	3.6	3	2.8	14	3.4

**Employment status**	managerial/professional	29	9.4	12	11.1	41	9.9
	
	middle management	80	26.0	47	**43.5^†^**	127	30.5
	
	skilled manual worker	39	12.7	14	13.0	53	12.7
	
	semi and unskilled manual worker	36	11.7	0	0	36	8.7
	
	state pensioner/retired	2	0.6	23	21.3	25	6.0
	
	off sick/disabled	13	4.2	3	2.8	16	3.8
	
	unemployed	99	32.1	7	6.5	106	25.5
	
	student	10	3.2	2	1.9	12	2.9

**Perceived socioeconomic status**	well below average	68	22.1	3	2.8	71	17.1
	
	slightly below average	92	29.9	19	17.6	111	26.7
	
	average levels of society	109	35.4	63	**58.3^†^**	172	41.3
	
	a little above average	36	11.7	22	**20.4^†^**	58	13.9
	
	much above average	3	1.0	1	0.9	4	1.0

^†^Chi square, p < 0.001

TSCs had some differences in terms of migration patterns. The number of participants who were born in Britain was higher in Group B (n = 28; 25.9%) than Group A (n = 12; 3.9%) (p < 0.001). The mean duration of stay in the UK for Group B (26.9 ± 13.9 years, range 4 to 54 years) was longer than that of Group A (13.3 ± 7.5 years, range 1 to 40 years); 43.8% of Group A stayed in the UK 'between 1-10 years' and 35.5% stayed 'between 11-20 years', the rates at Group B for the same periods were 16.7% and 9.3% respectively (p < 0.001). The main reasons for immigration at Group A were 'work related' (22.3%) and 'accompany and join to the family' (48.6%) the rates were similar for same reasons at Group B, 'work related' (21.2%) and 'accompany and join to the family' (56.2%) respectively. While all of the Group B participants had permanent residence (100%), only 79.1% of the Group A had permanent residence, (p < 0.001). TSCs' health promotion or risk behaviours and objective health status was given at Table 2. There was no significant difference in BMIs of Group B (26.4 ± 4.4) and Group A (25.5 ± 4.0) (p > 0.05). There were no significant differences between two groups in regular physical exercise, smoking and alcohol consumption. The rate of the presence of a chronic illness was significantly higher in Group B (36.1%) than Group A (24.0%) (p < 0.001).

**Table 2:** Health promotion or risk behaviours and objective health status of Turkish Speaking Communities

Health promotion or risk behaviours		Turkish Immigrants (Group A)		Turkish Cypriots (Group B)		Total	
		
		n	%	n	%	N	%
**Regular Exercise**	Yes	181	58.8	72	66.7	253	60.8
	
	No	127	41.2	36	33.3	163	39.2

**Smoking**	Yes	133	43.2	44	40.7	177	42.5
	
	No	175	56.8	64	59.3	239	57.5

**Alcohol intake**	Yes	46	14.9	16	14.8	62	14.9
	
	No	262	85.1	92	85.2	354	85.1

**Having chronic illness**	Yes	74	24.0	39	**36.1^†^**	113	27.2
	
	No	234	76.0	69	63.9	303	72.8

**Being disabled**	Yes	13	4.2	3	2.8	16	3.8
	
	No	295	95.8	105	97.2	400	96.2

**Body Mass Index ± SD**		25.5 ± 4.0		26.4 ± 4.4		25.7 ± 4.1	

^†^Chi square, p < 0.001

Nearly all the participants registered with a GP. Only 12 participants (3.9%) from Group A and one participant (0.9%) from Group B were not registered with a GP. Of participants, 78 (19.4%) reported that they were not using GP services. Of these, 38.5% (n = 30) reported that they had been using 'Turkish speaking private GP'. While 60.9% (n = 14) of Group B reported that they were using other private physician services in the UK, it was only 21.8% (n = 12) for Group A (p < 0.001). While 36.4% (n = 20) of Group A reported that they were using health care services in Turkey, only 8.7% (n = 2) Group B reported that they were using health care services in Turkey or Cyprus (p < 0.001). It was stated that Group A needed interpretation more often when using health care services (36.5%) than Group B (15%) (p < 0.001). Group A participants mostly used official interpreters and health advocates (21.6%) and their spouse (6.4%) for interpretation. A health services access index (HAI) was also developed. Mean HAI score was significantly higher in Group B compared to Group A (p < 0.0001) (Table 3).

**Table 3:** Access to health care services

Access to health care services		Turkish Immigrants (Group A)		Turkish Cypriots (Group B)		Total	
		
		n	%	n	%	n	%
Registered With GP	Yes	296	96.1	107	99.1	403	96.9
	
	No	12	3.9	1	0.9	13	3.1

Difficulty when getting referrals to a hospital	Yes	73	24.7	17	15.9	90	22.3
	
	No	223	75.3	90	84.1	313	77.7

Difficulty after being referred to a hospital	Yes	75	25.3	20	18.7	95	23.6
	
	No	221	74.7	87	81.3	308	76.4

Not using GP services	Yes	55	18.6	23	21.5	78	19.4
	
	No	241	81.4	84	78.5	325	80.6

Other type of health care services	Turkish speaking private GP	23	41.8	7	30.4	30	38.5
	
	Other private physicians	12	21.8	14	**60.9^†^**	26	33.3
	
	Visiting a physician in Turkey or Cyprus	20	**36.4^†^**	2	8.7	22	28.2

Need for interpretation for health care services	Yes	108	**36.5^†^**	16	15.0	124	30.8
	
	No	188	63.5	91	85.0	279	69.2

Interpreter for health care services	Spouse	19	17.6	1	6.3	20	16.1
	
	Siblings	5	4.6	0	0.0	5	4.0
	
	Children	9	8.3	6	37.4	15	12.1
	
	A family member	4	3.7	3	18.8	7	5.6
	
	A friend	7	6.5	1	6.3	8	6.6
	
	Official interpreter/health advocate	64	59.3	5	31.2	69	55.6

Access Index *	n	296		107		403	
	
	Mean	5.5 ± 1.3 (2.0-8.0)		6.2 ± 1.2 (2.0-8.0)		**5.7 ± 1.3^††^** (2.0-8.0)	

^†^p < 0.001, ^††^p < 0.0001, *A composite index derived from the individual Health Services Access items *(higher the index score, better the access to health care services)*

Table 4 compares Turkey originated and Cyprus originated communities on different aspects of health related QoL. Turkish Cypriots reported significantly better QoL and perceived health in overall QoL, perceived health and for psychological and environmental dimension scores than Turkish immigrants.

**Table 4:** Quality of Life dimensions of the participants

Country of Origin	Physical well-being	Psychological well-being	Social relationships	Environment	Perceived overall QoL	Self-rated Health
**Turkey**	15.6 ± 2.9	14.8 ± 2.6	14.6 ± 2.8	13.8 ± 2.2	3.04 ± 0.8	3.30 ± 1.0

**Cyprus**	15.8 ± 2.7	15.4 ± 2.2	15.1 ± 2.6	14.9 ± 1.8	3.46 ± 0.6	3.58 ± 0.8

**p**	ns*	p = 0.03	ns	p < 0.0001	p < 0.05	p = 0.008

*non significant

Young age (except for psychological dimension), male gender, being healthy (except for environmental dimension) and well-educated, home ownership, high socioeconomic status, better access to health care services, ability to communicate in English and better integration to community were significantly related to higher QoL scores in all of the scales of the WHOQOL. Smoking, alcohol intake, marital status, country of origin and reason for migration (except for environmental dimension) were not significantly related to any of the dimensions of QoL. There was significant relationship between age, socioeconomic status and education, access to health care services, community integration, obesity and physical well-being. Female gender, low income, being ill and obese were significantly related to psychological well-being; older age, living alone (single), non homeownership, low socioeconomic status were significantly related to social relationships dimension; poor educational and socioeconomic status, non homeownership and poor community integration were significantly related to environmental well-being dimension; low income and poor community integration were significantly related to perceived overall QoL; and being women, being ill and poor English speaking ability were significantly related to self-rated health (Table 5).

**Table 5:** WHOQOL domains and related factors

Related factors	WHOQOL domains					
	
	Physical well-being	Psychological well-being	Social relationships	Environment	Perceived overall QoL	Self-rated health
Age *(middle age)* *ref = younger age*	3.19 (1.12-9.09)	ns*	ns	ns	ns	ns

Age *(old)* *ref = younger age*	8.61 (2.51-29.51)	ns	2.63 (1.06-6.52)	ns	ns	ns

Gender *(female)*	ns	2.28 (1.24-4.19)	ns	ns	ns	1.98 (1.07-3.67)

Educational status *(low)*	ns	ns	ns	2.33 (1.07-5.09)	ns	ns

Perceived income *(poor)*	ns	2.64 (1.06-6.60)	0.48 (0.24-0.98)	3.59 (1.32-9.74)	6.07 (2.11-17.47)	ns

Coupling (marital) *(single)*	ns	ns	1.83 (1.05-3.19)	ns	ns	ns

Home ownership *(non)*	2.28 (1.10-4.75)	ns	1.86 (1.01-3.56)	2.41 (1.21-4.77)	ns	ns

Socioeconomic status *(low)*	ns	ns	1.82 (0.95-3.48)	ns	ns	ns

Reason for migration *(seeking job)*	ns	ns	ns	ns	ns	ns

Level of access to health care services *(poor access)*	2.73 (1.11-6.68)	ns	ns	ns	ns	ns

Objective health *(ill)*	8.64 (4.47-16.70)	1.95 (1.07-3.56)	ns	ns	ns	5.18 (2.63-10.10)

Community integration *(poor)*	2.22 (1.13-4.38)	ns	ns	2.79 (1.33-5.84)	5.18 (1.90-14.13)	ns

Body Mass Index *(BMI = 25.0-29.9)* *ref = BMI < 25.0*	ns	1.94 1.07-3.50	ns	ns	ns	ns

Body Mass Index *(BMI > 29.9)* *ref = BMI < 25.0*	ns	2.97 1.20-7.37	ns	ns	ns	ns

Ability to speak English *(poor)*	ns	ns	ns	ns	ns	1.91 (1.00-3.65)

Country of origin	ns	ns	ns	ns	ns	ns

*non significant

## Discussion

Migration has always been a characteristic of human society, and one that has probably always been pregnant with health challenges [2]. A significant number of Turkish speaking immigrants are living in London. However, there are only a few studies on their health care needs.

In our study, there were some differences among TSCs; Turkish immigrants were younger and less educated than Turkish Cypriots. The unemployment rate was higher in Turkish immigrants and Turkish Cypriots were more crowded in 'middle management'. The mean duration of stay in the UK was longer for Turkish Cypriots and all of them had permanent residence. Turkish Cypriots had come earlier and were more settled than immigrants from mainland Turkey. They have a colonial connection with Britain so their ties to Britain are stronger [16].

Although most of the participants registered with a GP, nearly one fifth reported that they were not using GP services. Language barriers made it difficult to access health care services for minority ethnic groups. Migrants often used family members and children as interpreters. Cinar had investigated primary health care needs of TSCs (n = 129) in London; more than half of the respondents stated that they were experiencing communication problems and needed interpretation when using health care services (n = 72, 55.8%) [5]. In our study, Turkish immigrants needed interpretation more often when using health care services than Turkish Cypriots and they mostly used official interpreters, health advocates and their spouse for interpretation. The need for interpreter was lower in Turkish Cypriots than that of Turkish immigrants (36.5% vs. 15%).

QoL scores of Turkish immigrants and Turkish Cypriots were higher than those obtained from the Turkish population living in Turkey [18]. These results might be attributed to the higher educational status of the respondents of this study compared to the educational status of the population in Turkey. On the other hand, Turkish Cypriots were more likely to have higher scores in overall perceived QoL, perceived health, physical well-being and social relationship dimension scores than Turkish immigrants. However, none of the dimensions were sensitive to the country of origin in the logistic regression analyses.

It was found in literature that older age, female gender, having any chronic disease, having a low educational and socioeconomic status worsen QoL scores. Older age showed a special effect of physical well-being which would be logical since older age is the age of chronic diseases. Bayram et al reported that those who immigrated at younger ages had a better QoL in Turkish immigrants in Sweden. [19] A number of international normative QoL data [20-22] including Turkish normative data [18,23] also indicated the negative effect of age on QoL.

Turkish immigrant studies from Sweden and the Netherlands also reported that there was a gender difference in QoL in favour of males [19,24]. In our study, we found that there was a significant relationship between gender and psychological well-being and self-rated health and women had lower scores. However, other studies found lower scores almost in all domains of WHOQOL for women [19-22]. It was reported that men had significantly higher physical well-being scores than women, whereas some of the sub-domains of psychological well-being were not distinctive among men and women [23].

Only the environmental well-being was affected from low educational status, perceived income and home ownership. It is known this domain is much more sensitive to socioeconomic variables than other factors and also could not discriminate ill and well persons [15,17]. Our results showed that low income was significantly related to psychological and environmental well-being, social relationships, and overall QoL. No significant association was found between marital status and all four domains. Our results were consistent with some other previous studies [25,26].

The results showed that not owning a home affected physical, social and environmental well-being negatively. Owning a property is an important economic and social indicator. Having a lower socioeconomic status and physical and psychological well-being, social relationships, self-rated health and being unhealthy were significantly related. Low socioeconomic class was significantly related to self-rated health [27-29].

Migrants appear to be more exposed to physical or mental health problems than the rest of the population due to their vulnerable situation and to cultural obstacles in host countries. These health risks increase when compounded by limited access to health care services [1-3]. Poor access to health care services was affected only by the physical well-being negatively. Leavey et al underlined the different nature and importance of the stigma of mental illness in the TSCs [10]. Turkish patients could not easily reveal their mental illness and dislike having someone to translate deeply personal and intimate thoughts and experiences. They are likely to reject or avoid if possible, contact with non Turkish doctors for their mental health problems. May be they could not express themselves fairly based on these cultural aspects and health beliefs.

Antonovsky pointed out the importance of the psychological, social, and cultural resources that people can use and the role of "sense of coherence" to stay healthy [30]. It is also an important factor in a migrant's capacity to cope with stress and improve QoL during the early adaptation. Many organizations and programs in Europe supported research projects and policy works to help ensure that migration and integration can be managed in a socially just and equitable manner [31,32]. In our study, community integration was found to be associated with the economic power. It was the only variable showing significant relationship with the perceived overall QoL. We found that obesity was significantly related to the psychological well-being. There are some studies focusing on the negative effect of overweight on mental well-being [33-35].

On the other hand, ability to speak English was significantly related to all dimensions in the univariate analysis whereas it was not related to any of the dimensions in the multivariate analysis. These contradicting results suggest that ability to speak English was represented by any other variables such as education, income etc. that were already included in the regression models.

This study has some limitations. First, our results cannot be generalized to the Turkish community living in London due to lack of records of Turkish immigrants living in London. We had to design a mixed sampling method cluster sampling and snowball sampling which cannot be regarded as a probability sampling approach. A second limitation was the measurement of the accessibility of the health care services which was not based on pre-validated measures. A number of recently validated instruments and methods are available for the measurement of the performance of the health care services and health promotion [36-41]. We did not use any of them as they are very long and our respondents will have time constraints. Instead, we followed the main concepts and domains of these instruments while developing our semi-structured questionnaire for the assessment of accessibility of health care. However, our questionnaire needs to be validated.

The results of this study not only demonstrated how the health and well-being of members of the Turkish speaking community living in London were affected by social aspects of their lives but also point to the need for urgent action by local statutory services to address the social issues raised, such as language barriers and integration. Nevertheless, QoL of the Turkish community living in London has a moderate level of health related QoL. The good thing is the indifference of the physical and social well-being scores of the Turkey originated and Cyprus originated Turkish community.

Providing culturally competent care and interpretation services and advocacy may improve the accessibility of the health care services. We anticipate that sharing the results of this study with the public authorities will contribute to the policy making process.

## Competing interests

The authors declare that they have no competing interests.

## Authors' contributions

**KT **conceived of the study, performed the literature review, contributed to design, conducted and supervised data collection and drafted the manuscript. **EE **contributed to design, conducted and supervised statistical analysis and contributed to the manuscript. **IS **contributed to design and conducted the statistical analysis. **EB **contributed to design, coordinated data collection and contributed to the manuscript. **ES **drafted and edited the manuscript. All authors have read and approved the final manuscript.

## Funding

This study was supported by the Social Action for Health, London (SAfH) and funded by the Scientific and Technological Research Council of Turkey (TUBITAK).

## Supplementary Material

Additional file 1 **Identifying Challenges to Quality Of Life-Questionnaire 2010**. A structured questionnaire with 44 items in five subcategories and 26 items WHOQOL BREF developed by the World Health Organization. Click here for file
