# Supplementary material for: Challenges in access to health services and its impact on quality of life: a randomised population-based survey within Turkish speaking immigrants in London
Source: Health Qual Life Outcomes. 2012 Jan 26;10:11. doi: 10.1186/1477-7525-10-11 (PMC3275503; doi:10.1186/1477-7525-10-11)
Supplement: Additional file 1 — Identifying Challenges to Quality Of Life-Questionnaire 2010. A structured questionnaire with 44 items in five subcategories and 26 items WHOQOL BREF developed by the World Health Organization. [file 1477-7525-10-11-S1.PDF]

Social Action for Health

Identifying Challenges to Quality Of Life - Questionnaire 2010

**Name of Client**	**:**
**Name of interviewer :**	

INTRODUCTION

Thank you very much for agreeing talk to me about your experiences when accessing health care services. We hope that the results will make an impact on your overall life quality. I'd like to start by saying that everything you say to me will kept confidential to two of us that are conducting these surveys a report will be compiled and no names will be mentioned. Nothing will be reported to anyone else in a way that will allow you to be identified.

I'd like to ask you about several different things. I reckon this will take about 15 minutes. If you do not want to answer any of the questions it is all right. Is that OK?

ABOUT YOU

1. **What is your gender?** male ☐ female ☐

2. **What is you date of birth?** _____ / _____ / ________
   day / month / year

3. **What is the highest education you received?**
   none at all ☐ primary school ☐ secondary school ☐ tertiary ☐

4. **What is your marital status?**
   single ☐ married ☐ living with partner ☐ separated ☐ divorced ☐ widowed ☐

5. **How do you describe your household type?**
   ____ adult/adults, ____ child/children, _____other person (please state ____________________)

6. **How do you describe your employment status?** (*please see the table at last page about explanation of jobs*)
   1. Managerial/professional ☐ 5. State pensioner/retired ☐
   2. Middle management ☐ 6. Off sick/disabled ☐
   3. Skilled manual worker ☐ 7. Unemployed ☐
   4. Semi and unskilled manual worker ☐ 8. Student ☐
   (*please go to question 16 if you cross boxes 5, 6, 7 or 8*)

7. **Are you householder of your family? If not how do you describe employment status of your family's chief economic supporter?**
   1. Managerial/professional ☐ 5. State pensioner/retired ☐
   2. Middle management ☐ 6. Off sick/disabled ☐
   3. Skilled manual worker ☐ 7. Unemployed ☐
   4. Semi and unskilled manual worker ☐

8. **Would you mind if I ask you to compare your income with the community?***	well below average	slightly below average	average levels of society	a little above average	much above average
	1	2	3	4	5

9. **In which sector you are working?** service ☐ industry ☐ agriculture ☐

10. **For how long you have been working in this sector?** For ________ years and ______ months.

11. **For how long you have been working in this workplace?** For ________ years and ______ months.

	not at all	slightly	moderately	very much	extremely
**12. Does your job fulfill you?**	1	2	3	4	5
**13. Do you feel valued and respected in your workplace?**	1	2	3	4	5

	never	rarely	sometimes	often	always
**14. Are you suffering from workplace stress?**	1	2	3	4	5
**15. How often do you get help and support from your co-workers?**	1	2	3	4	5

16. **In which country did you born?** _____________

17. **How would you describe your ethnic group?**

**A. White**<br>British ☐<br>Irish ☐<br>Any other White background ☐<br>(*please state* _______________)<br><br>**B. Mixed**<br>White and Black Caribbean ☐<br>White and Black African ☐<br>White and Asian ☐<br>Any other Mixed background ☐<br>(*please state* _______________)	**C. Asian or Asian British**<br>Indian ☐<br>Pakistani ☐<br>Bangladeshi ☐<br>Any other Asian background ☐<br>(*please state* ______________)<br><br>**D. Black or Black British**<br>Black Caribbean ☐<br>Black African ☐<br>Any other Black background ☐<br>(*please state* ______________)	**E. Chinese or any other ethnic group**<br>Chinese ☐<br>Any other ethnic group ☐<br>(*please state* ____________)<br><br>**F. Turkish Speaking Community**<br>Turkish ☐<br>Turkish Cypriot ☐

18. **How long have you lived in Britain?** For ________ years. / (☐ Born in UK)

	not at all	slightly	moderately	very much	extremely
**19. Do you have sense of coherence within society?**	1	2	3	4	5

20. What is the type of your housing tenure?
a. Rented ☐; local authority rented ☐ rented furnished ☐ other rented unfurnished ☐
housing association or RSL ☐ rent free ☐
b. Owner occupied ☐; with mortgage ☐ owned outright ☐

21. If you are an immigrant what was your main reason for immigration to Britain?
work related ☐ accompany/join ☐ formal study ☐ other ☐ (please state:____________)
not an immigrant ☐ (*please go to the question 23*)

22. What is your residential status in the Britain?
permanent residence ☐ temporary residence ☐ refugee ☐ asylum seeker ☐

**23. Please rate level of your English?**	can't speak English	not at all influential	somewhat influential	very influential	extremely influential
	1	2	3	4	5

24. Are you currently ill? Yes ☐ No ☐

25. If something is wrong with your health what do you think it is? _______________illness/ problem

26. Do you have any serious chronic illness? Yes ☐ No ☐ (*please state if yes* __________________)

27. Are you disabled? Yes ☐ No ☐

28. Do you consider yourself disabled? Yes ☐ No ☐

29. Are you registered with a GP? Yes ☐ No ☐ (*if you cross No please go to question 37*)

30. Do health and social care professionals make regular home visits? Yes ☐ No ☐
(for women, children and elderly)

31. Do you experience difficulties when you are referring to a hospital? Yes ☐ No ☐

32. Do you experience difficulties after you referred to a hospital? Yes ☐ No ☐

	very difficult	difficult	neutral	easy	very easy
**33. How would you rate the level of financial difficulty in accessing health services?**	1	2	3	4	5
**34. How would you rate the level of cultural obstacles in accessing health services?**	1	2	3	4	5
**35. How easily do you communicate with your health professionals in English?**	1	2	3	4	5

36. Who interprets for you when using the GP service?
nobody ☐ spouse ☐ siblings ☐ my children ☐
a family member ☐ a friend ☐ interpreter/ health advocate ☐

37. Do you get primary health care in another way although you are registered with GP? Yes ☐ No ☐
private GP ☐ other private doctor ☐ (please state: ____________________)
I am going to doctor in another country ☐

38. If you are not registered to a GP how do you access to health services?
private GP ☐ other private doctor ☐ (please state: ____________________)
I am going to doctor in another country ☐ I did not ever go to a doctor ☐

39. Your weight ______ kg / your height _______ ft ________ in (or________ cm)

40. How do you perceive your body image by weight and height?
By weight; underweight ☐ normal weight ☐ overweight and obese ☐
By height; short ☐ medium ☐ tall ☐

41. Do you walk or do some other form of activity for exercise? Yes ☐ No ☐
If yes please state type and duration of exercise. Type of exercise: ____________________
_____minutes in a day usually, ______ days per week usually.

42. Do you smoke? Yes ☐ No ☐ **If yes how many cigarettes per day do you usually smoke?**
Please write the number on the line and circle one response. _____ cigarettes per day.

10 or less ☐ 11 to 20 ☐ 21 to 30 ☐ 31 to 40 ☐ 41 or more ☐

43. How soon after you wake up do you smoke your first cigarette?
within five minutes ☐ 6-30 minutes ☐ more than 30 minutes ☐

44. Do you drink alcohol? Yes ☐ No ☐ **If yes please specify how many units per week.** (e.g. glass wine= 2, pint of beer= 2, shot of whiskey= 1 unit) _____ units per day, _____days per week usually.
Total ______ units per week.

WHOQOL-BREF

Instructions: This assessment asks how you feel about your quality of life, health, or other areas of your life. **Please answer all the questions.** If you are unsure about which response to give to a question, **please choose the one** that appears most appropriate. This can often be your first response.

Sample question: Please keep in mind your standards, hopes, pleasures and concerns. We ask that you think about your life **in the last two weeks.** For example, thinking about the last two weeks, a question might ask:

**SAMPLE QUESTION**	Not at all	Not much	Moderately	A great deal	Completely
Do you get the kind of support from others that you need?	1	2	3	4	5

You should circle the number that best fits how much support you got from others over the last two weeks. So you would circle the number 4 if you got a great deal of support from others as follows.

**SAMPLE QUESTION**	Not at all	Not much	Moderately	A great deal	Completely
Do you get the kind of support from others that you need?	1	2	3	(4)	5

You would circle number 1 if you did not get any of the support that you needed from others in the last two weeks.

Please read each question, assess your feelings, and circle the number on the scale for each question that gives the best answer for you.

		Very poor	Poor	Neither poor nor good	Good	Very good
**1**	How would you rate your quality of life?	1	2	3	4	5

		Very dissatisfied	Dissatisfied	Neither satisfied nor dissatisfied	Satisfied	Very satisfied
**2**	How satisfied are you with your health?	1	2	3	4	5

The following questions ask about **how much** you have experienced certain things in the last two weeks.

		Not at all	A little	A moderate amount	Very much	An extreme amount
**3**	To what extent do you feel that physical pain prevents you from doing what you need to do?	1	2	3	4	5
**4**	How much do you need any medical treatment to function in your daily life?	1	2	3	4	5
**5**	How much do you enjoy life?	1	2	3	4	5
**6**	To what extent do you feel your life to be meaningful?	1	2	3	4	5
**7**	How well are you able to concentrate	1	2	3	4	5
**8**	How safe do you feel in your daily life?	1	2	3	4	5
**9**	How healthy is your physical environment?	1	2	3	4	5

The following questions ask about **how completely** you experience or were able to do certain things in the last two weeks.

		Not at all	A little	Moderately	Mostly	Completely
**10**	Do you have enough energy for everyday life?	1	2	3	4	5
**11**	Are you able to accept your bodily appearance?	1	2	3	4	5
**12**	Have you enough money to meet your needs?	1	2	3	4	5
**13**	How available to you is the information that you need in your day-to-day life?	1	2	3	4	5
**14**	To what extent do you have the opportunity for leisure activities?	1	2	3	4	5

		Very poor	Poor	Neither poor nor good	Good	Very good
**15**	How well are you able to get around?	1	2	3	4	5

Social Action for Health

Identifying Challenges to Quality Of Life - Questionnaire 2010

The following questions ask you to say how **good or satisfied** you have felt about various aspects of your life over the last two weeks.

		Very dissatisfied	Dissatisfied	Neither satisfied nor dissatisfied	Satisfied	Very satisfied
**16**	How satisfied are you with your sleep?	1	2	3	4	5
**17**	How satisfied are you with your ability to perform your daily living activities?	1	2	3	4	5
**18**	How satisfied are you with your capacity for work?	1	2	3	4	5
**19**	How satisfied are you with yourself?	1	2	3	4	5
**20**	How satisfied are you with your personal relationships?	1	2	3	4	5
**21**	How satisfied are you with your sex life?	1	2	3	4	5
**22**	How satisfied are you with the support you get from your friends?	1	2	3	4	5

The following questions ask you to say how **good or satisfied** you have felt about various aspects of your life over the last two weeks.

		Very dissatisfied	Dissatisfied	Neither satisfied nor dissatisfied	Satisfied	Very satisfied
**23**	How satisfied are you with the conditions of your living place?	1	2	3	4	5
**24**	How satisfied are you with your access to health services?	1	2	3	4	5
**25**	How satisfied are you with your transport?	1	2	3	4	5

The following question refers to **how often** you have felt or experienced certain things in the last two weeks.

		Never	Seldom	Quite often	Very often	Always
**26**	How often do you have negative feelings such as blue mood, despair, anxiety, depression?	1	2	3	4	5

THANK YOU FOR YOUR HELP
